# Investigating the Immunomodulatory Potential of Dental Pulp Stem Cell Cultured on Decellularized Bladder Hydrogel towards Macrophage Response In Vitro

**DOI:** 10.3390/gels8030187

**Published:** 2022-03-18

**Authors:** Huynh-Quang-Dieu Nguyen, Chen-Yu Kao, Chien-Ping Chiang, Yu-Han Hung, Chun-Min Lo

**Affiliations:** 1 Graduate Institute of Applied Science and Technology, National Taiwan University of Science and Technology, Taipei 10607, Taiwan; d10822805@mail.ntust.edu.tw; 2 Graduate Institute of Biomedical Engineering, National Taiwan University of Science and Technology, Taipei 10607, Taiwan; 3 Department of Biomedical Engineering, National Defense Medical Center, Taipei 11490, Taiwan; 4 Department of Biochemistry, National Defense Medical Center, Taipei 11490, Taiwan; cppchiang@gmail.com; 5 Department of Dermatology, Tri-Service General Hospital, Taipei 11490, Taiwan; 6 Department of Biomedical Engineering, National Yang Ming Chiao Tung University, Taipei 11221, Taiwan; yuhanhung.be05@nycu.edu.tw (Y.-H.H.); chunmin.lo@nycu.edu.tw (C.-M.L.)

**Keywords:** bladder extracellular matrix, decellularization, hydrogel, dental pulp stem cell, immunomodulation

## Abstract

Mesenchymal stem cells (MSCs) possess immunomodulatory properties and capacity for endogenous regeneration. Therefore, MSC therapy is a promising treatment strategy for COVID-19. However, the cells cannot stay in the lung long enough to exert their function. The extracellular matrix from porcine bladders (B-ECM) has been shown not only to regulate cellular activities but also to possess immunoregulatory characteristics. Therefore, it can be hypothesized that B-ECM hydrogel could be an excellent scaffold for MSCs to grow and could anchor MSCs long enough in the lung so that they can exhibit their immunomodulatory functions. In this study, ECM degradation products and a co-culture system of MSCs and macrophages were developed to study the immunomodulatory properties of ECM and MSCs under septic conditions. The results showed that B-ECM degradation products could decrease pro-inflammatory and increase anti-inflammatory cytokines from macrophages. In an in vivo mimicking co-culture system, MSCs cultured on B-ECM hydrogel exhibited immunomodulatory properties at both gene and protein levels. Both B-ECM degradation products and MSC conditioned medium supported the wound healing of alveolar epithelial cells. The results from the study could offer a basis for investigation of immunomodulation by ECM and MSCs before conducting in vivo experiments, which could later be applied in regenerative medicine.

## 1. Introduction

Almost every country in the world has suffered from the COVID-19 pandemic. COVID-19 primarily attacks the lungs and respiratory tract through dysregulation of the immune system which causes cytokine release syndrome (CRS) [1]. Currently, there is no effective treatment to mitigate the syndrome and help to regenerate the damaged tissue [2]. There is increasing clinical evidence that, in addition to standard treatments, it is imperative for critically ill and elderly patients, in particular, to receive other adjuvant therapies to lower mortality rate and ameliorate recovery [3]. One of the emerging supplementary therapies which has been accepted by clinics or in clinical trials is the use of mesenchymal stem cells (MSCs) [4].

The reason why MSCs are beneficial in treating COVID-19 is that they possess anti-inflammatory/immunomodulatory ability and can activate endogenous regeneration [5]. Several studies have reported the anti-inflammatory effects of MSCs in animal models resulting from a significant reduction in pro-inflammatory cytokines, such as TNF-α, and an increase in anti-inflammatory cytokines, such as IL-10 [6,7]. Presently, the majority of clinical trials employ intravenous infusion as the main method to transplant MSCs into patients [8]. However, in a phase I clinical trial conducted by Armitage et al., the authors found that, after the stem cells were injected into the body intravenously, they only stayed in the lungs for the first half hour and then gradually migrated to the liver [9]. The inability of stem cells to stay in the lung long enough may affect the therapeutic effects of stem cells for COVID-19 treatment. Attempted solutions to this problem have included adjusting the schedule or mode of administration. However, the outcome of these attempts have not always been promising [10,11]. Therefore, other strategies to enable stem cells to stay longer in the lungs need to be considered.

The extracellular matrix (ECM) is a three-dimensional lattice structure secreted and maintained by the cells, and is also known as the non-cellular component of tissues and organs [12]. ECM components include many macromolecules, such as collagen, glycoproteins and glycosaminoglycan, which not only constitute a physical scaffold for cellular adhesion, but also regulate cellular proliferation, migration and differentiation [13,14]. When implanted into the body, ECM scaffolds trigger a favorable host innate immune response, specifically, the macrophage response prior to constructive remodeling outcomes. Upon implantation, the ECM degrades and releases chemoattractant, antimicrobial, and mitogenic peptides, growth factors, and extracellular vesicles. These biogenic substances attune the behavior of responding immune cells toward a regulatory anti-inflammatory phenotype [15]. When exposed to the degradation products of the ECM, macrophages exhibit a distinctive phenotype associated with suppression of inflammation and high antigen-presenting capabilities. Furthermore, ECM materials express their immunomodulatory properties, not only by directly influencing the macrophage phenotype but also through paracrine effects, mediating macrophage crosstalk with endogenous stem/progenitor cells [16]. The ability of ECM scaffolds to encourage a pro-regenerative microenvironment has been explored in many in vitro and preclinical studies. A study by Badylak et al. indicated that, compared to cellular autografts, decellularized urinary bladder matrix (UBM) allografts promoted anti-inflammatory macrophage polarization and reduced fibrotic response to abdominal wall implants in rats [17]. In another study, a commercial UBM (Micro Matrix) was used to investigate the innate and adaptive immune response in a mouse model. The results indicated that MHC-II+ cells from UBM-treated mice were over 90% anti-inflammatory (CD206+), suggesting that the materials shifted anti-inflammatory signaling between innate and adaptive immune compartments [18]. These results underline the immunomodulatory properties, as well as the ability to promote functional tissue replacement, of acellular biological scaffolds.

**Scheme 1:**
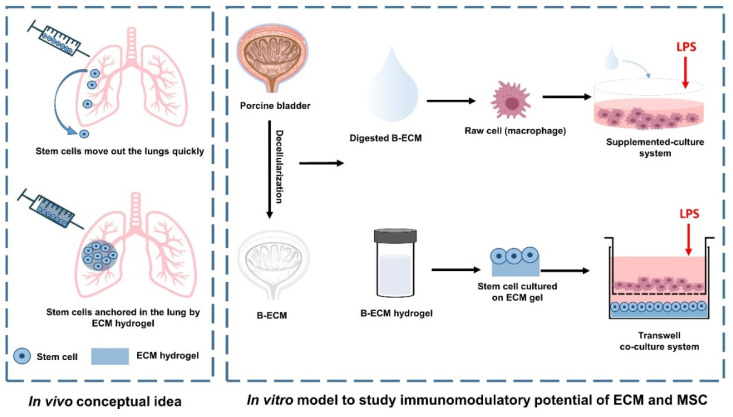
Experiment idea and design.

According to the literature mentioned above, ECM materials possess promising immunomodulatory properties. From another perspective, ECM, in the form of a hydrogel, has been shown to support cell attachment, proliferation and differentiation [19]. As a result, it can be hypothesized that ECM hydrogel from decellularized porcine bladder (B-ECM) can be used as a scaffold to anchor stem cells to enable them to stay longer in the lung to exert their immunomodulatory effects on immune cells. This could be further exploited as an adjuvant therapy for cytokine-induced lung injuries, or B-ECM degradation products themselves could be promising materials to regulate the response of immune cells (Scheme 1).

To the best of our knowledge, there has, to date, been no study investigating the possibility of using B-ECM hydrogel as a scaffold for culturing dental pulp stem cells (DPSCs) in relation to their immuno-regulatory effects towards macrophage response. Therefore, the objectives of this study were to preliminarily investigate: (1) the ability of bladder ECM (B-ECM) hydrogel to support the growth of DPSCs, (2) the immunomodulatory effect on the pulmonary macrophage RAW 264.7 of digested B-ECM, and (3) of DPSCs cultured on B-ECM hydrogel using an “in vitro mimicking” co-culture system. The expression profiles of cytokines, such as TNF-α (pro-inflammatory cytokine) and IL10 (anti-inflammatory cytokine), were analyzed at both gene and protein level. The results from the study cast light onto the immunomodulatory potential of ECM materials as promising candidates for application in regenerative medicine.

## 2. Results

### 2.1. Decellularization Confirmation

**Figure 1:**
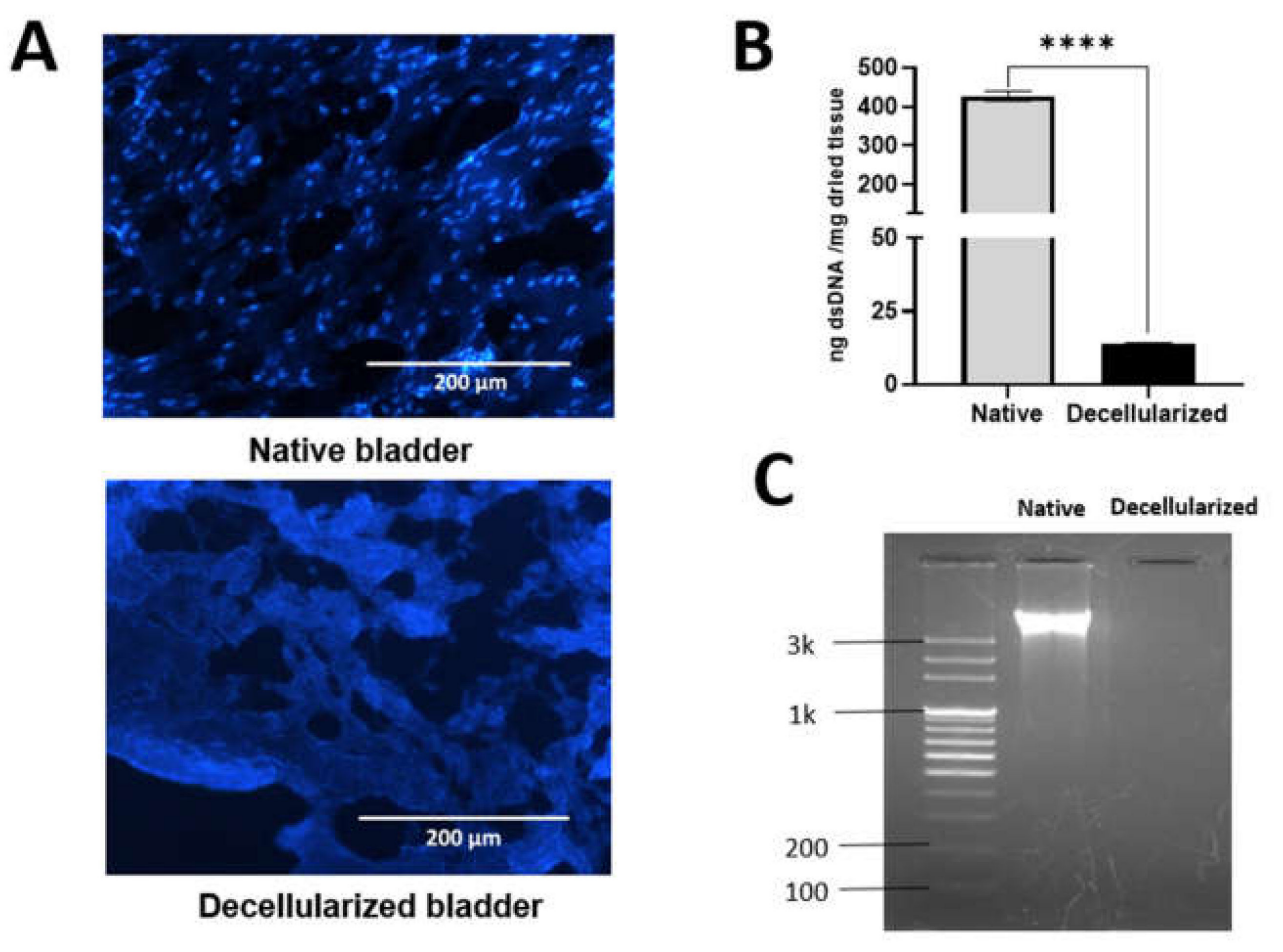
Decellularization efficiency. (**A**) DAPI stain (scale bar 200 µm). (**B**) dsDNA content by Picogreen assay (*n* = 3). (**C**) DNA electrophoresis. **** denotes a statistically significant difference (*p* < 0.0001).

After treatment with 1% SDS and 10% FCS, porcine bladders were effectively decellularized with no nuclei being detected in DAPI staining and less than 50 ng dsDNA per 1 mg dried tissue recorded. DNA fragments were also less than 100 base pairs in length using an electrophoresis test (Figure 1). The results were in accordance with our previous studies [20], confirming the stability of the decellularization procedure.

### 2.2. Mesenchymal Stem Cell Verification

**Figure 2:**
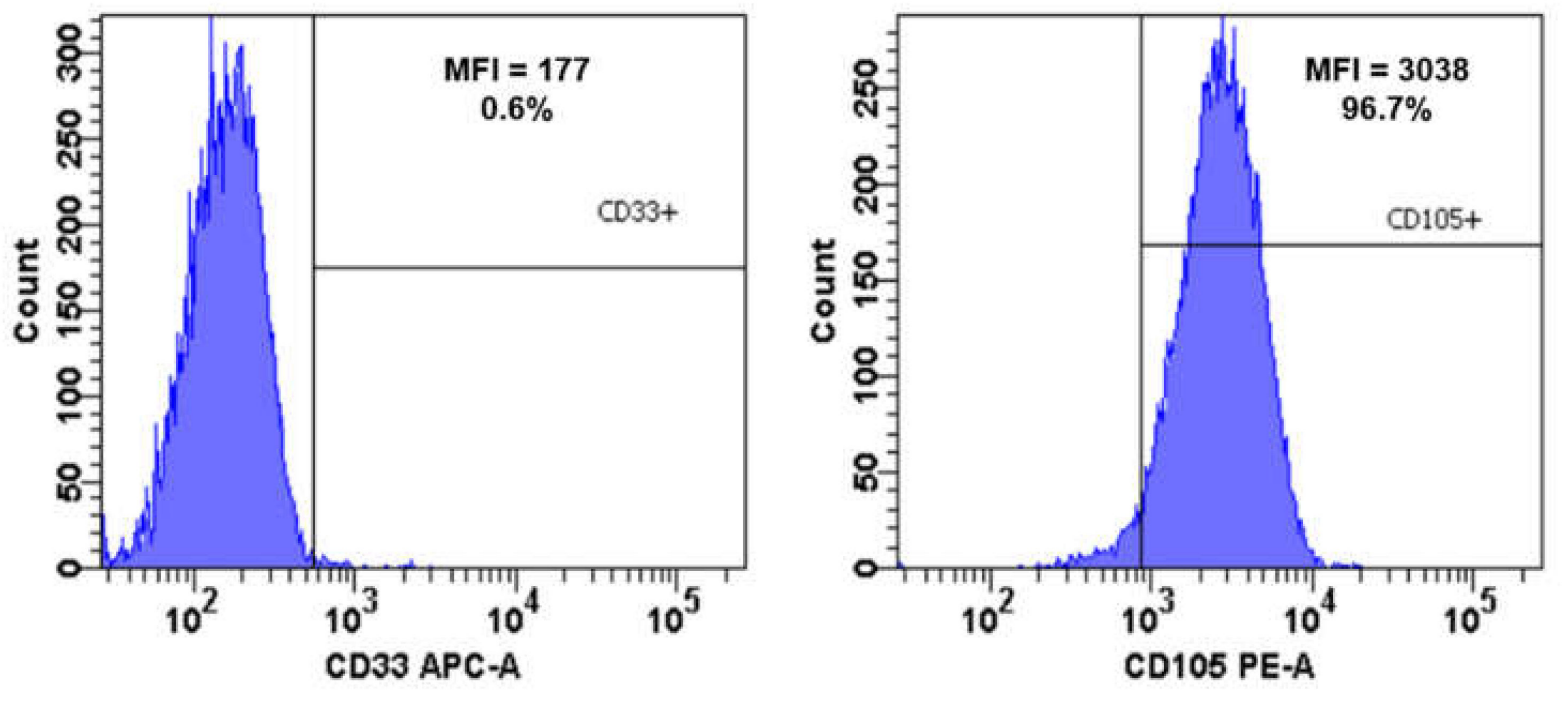
Flow cytometric analysis of mesenchymal stem cell surface marker CD33 (negative) and CD105 (positive).

The expression of mesenchymal stem cell surface markers was studied using flow cytometry (Figure 2). The results indicated high expression of CD105, a positive surface stem cell marker with a mean fluorescent intensity (MFI) of 3038, compared to low expression of CD33, a negative surface stem cell marker with an MFI of 177. Positive cells accounted for 96.7% of the total population, confirming the stemness of the DPSC source.

### 2.3. B-ECM Hydrogels In Vitro Degradation

**Figure 3:**
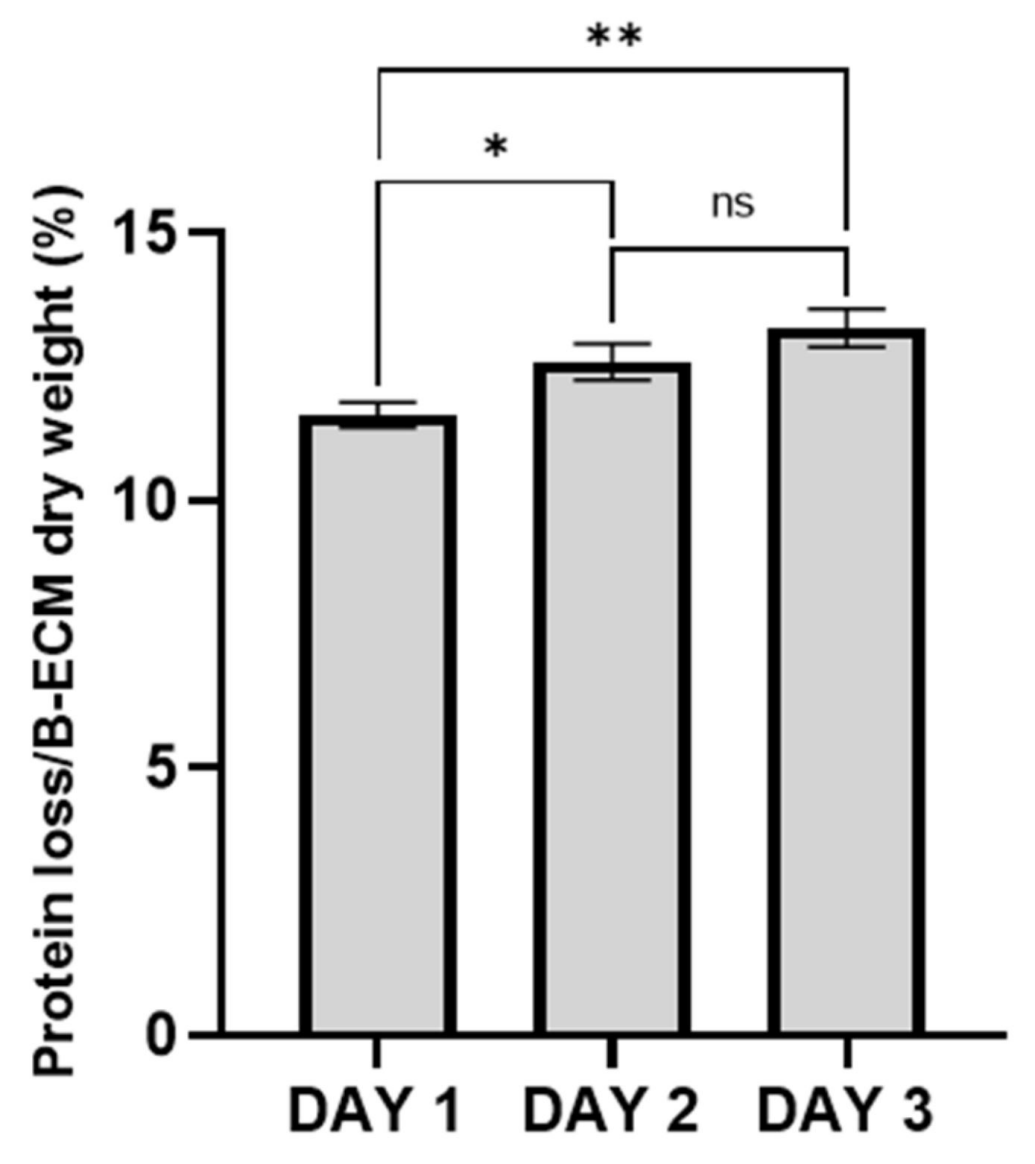
In vitro degradation of B-ECM hydrogel after 3 days (*n* = 3) with * (*p* ≤ 0.05), ** (*p* ≤ 0.01).

The degradation of B-ECM in PBS solution was studied using the accumulative total protein released method. As shown in Figure 3, in the first day there was approximately 11% protein release inside the PBS. However, after 2 and 3 days, the degradation rate of B-ECM hydrogel seemed to slow down with no significant difference between day 2 and 3.

### 2.4. DPSC Proliferation on Hydrogel Surface

**Figure 4:**
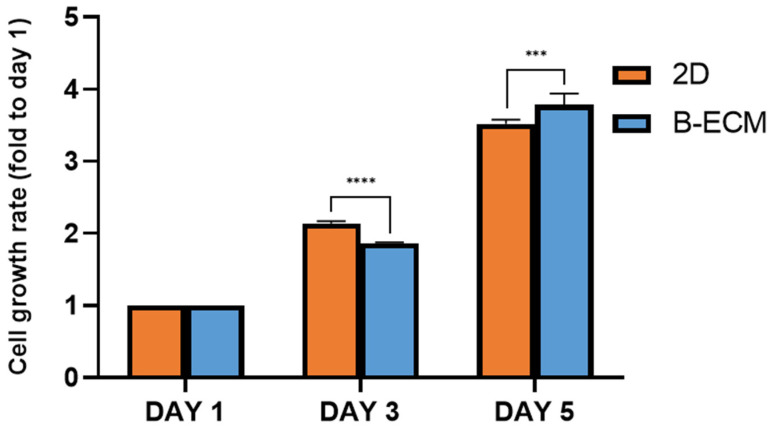
DPSCs grown on different substrates over 5 days, measured by Alamar blue assay (*n* = 3). *** *p* ≤ 0.001, and **** *p* ≤ 0.0001.

Alamar blue results showed that B-ECM hydrogel supported the growth of DPSCs better than the 2D control (Figure 4). In the first 3 days, DPSCs grew faster on the 2D stiff substrate. However, at day 5, B-ECM was significantly superior to the 2D group in DPSC cell growth rate, indicating the beneficial effect of the ECM components on cell proliferation. There was no difference in the morphology of DPSCs cultured on 2D or hydrogel substrate. The optical images can be found in the Supplementary Materials (Figure S1).

### 2.5. Macrophage Response Assays

**Figure 5:**
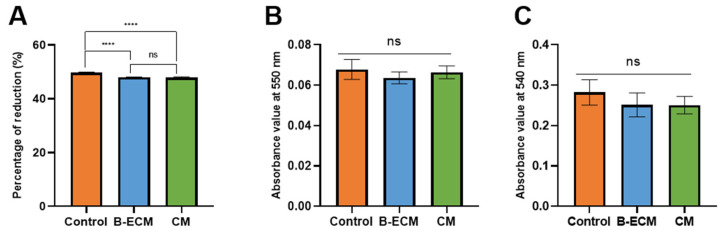
Macrophage response assay assessed by Alamar blue assay (**A**), NBT test (**B**) and NR test (**C**) (*n* = 4) when RAW cells were cultured in SM (control) with digested B-ECM or CM with ns being no significant and **** *p* ≤ 0.0001.

In supplemented-culture groups, adding digested B-ECM and CM to the standard medium did not strongly affect the viability of RAW cells as shown in Figure 5A. In terms of macrophage function, NBT was the assay used for determining phagocytic activity and NR indicates the pinocytic activity of macrophages. As shown in Figure 5B,C, culture of RAW cells with digested B-ECM and CM supplement did not alter the functional state of macrophages.

### 2.6. Immunomodulatory Effect of Digested B-ECM towards RAW Cells

**Figure 6:**
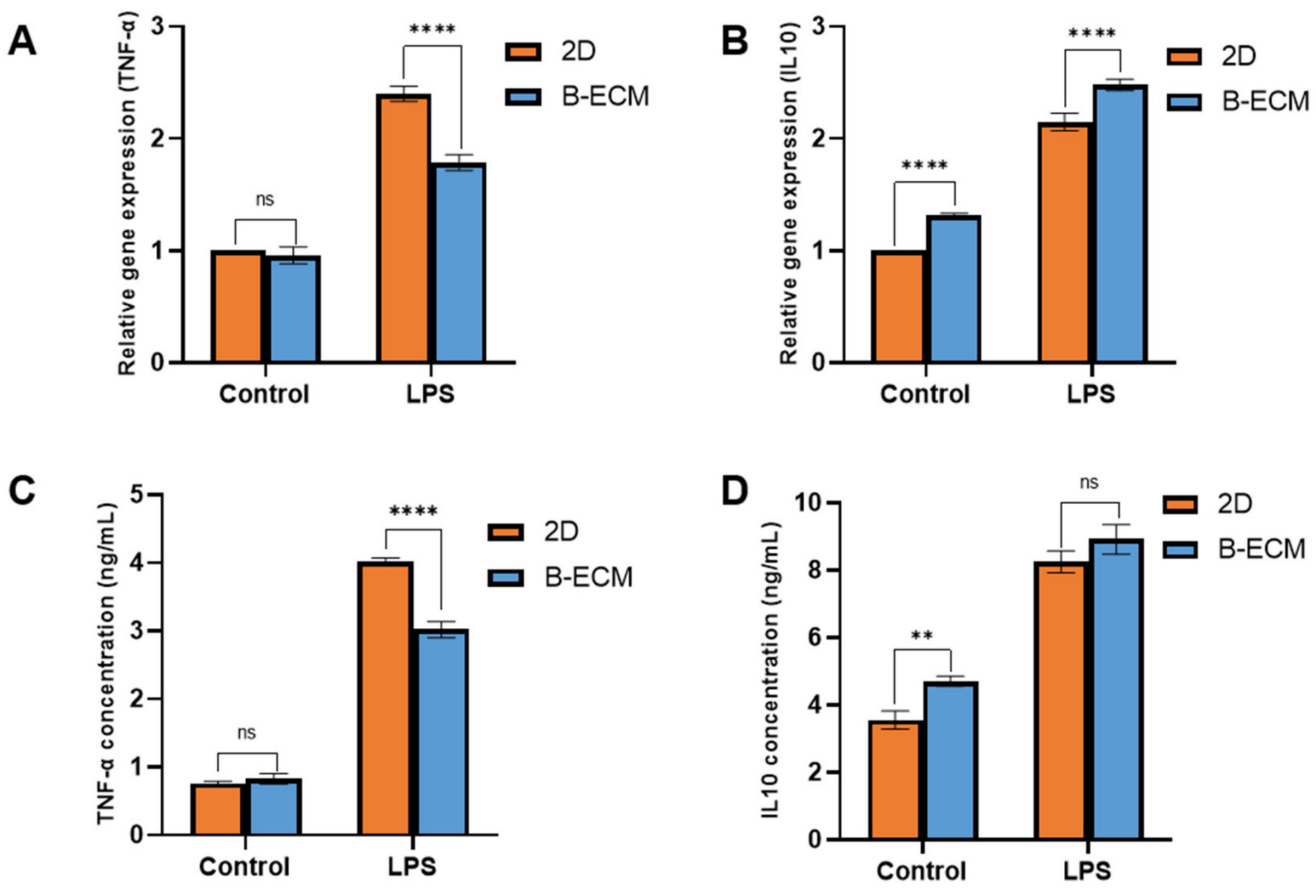
Immunomodulatory effect of digested B-ECM towards RAW cells under aseptic and septic conditions. Relative expression of TNF-α (**A**) and IL10 (**B**) genes by qPCR. TNF-α (**C**), IL-10 (**D**) concentration in cell culture supernatant by ELISA assay with ns being no significant, ** *p* ≤ 0.01, and **** *p* ≤ 0.0001.

The immunomodulatory effect of digested B-ECM towards RAW cells was studied by qPCR and ELISA assay. From Figure 6A,B, at gene level, under the unstimulated condition, digested B-ECM did not have a profound effect on TNF-α gene expression, but significantly upregulated the expression of the IL10 gene. Under the stimulated condition, the immunomodulatory ability of digested B-ECM was striking, with downregulation of TNF-α and upregulation of the IL10 gene compared to the 2D control. In protein measurement (Figure 6B,C), RAW cells with digested B-ECM exhibited the same level of TNF-α cytokines but released more IL10 under the aseptic condition compared to the control. Under the inflammatory condition, B-ECM groups greatly reduced the concentration of the pro-inflammatory cytokine TNF-α and promoted the release of the anti-inflammatory cytokine IL10, although not significantly.

### 2.7. Immunomodulatory Effect of DPSC on B-ECM Hydrogel towards RAW Cell under “In Vivo Mimicking” Co-Culture System

**Figure 7:**
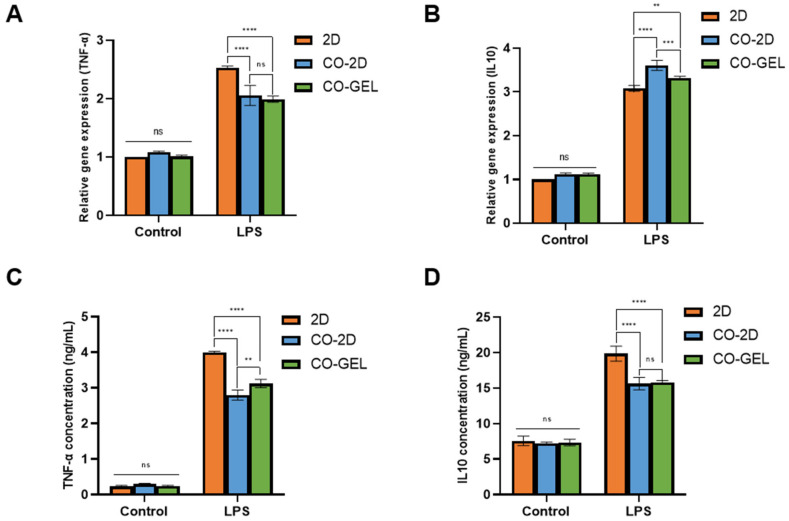
Immunomodulatory effect of DPSC on B-ECM hydrogel towards RAW cells under co-culture system under aseptic and septic conditions. Relative expression of TNF-α (**A**) and IL10 (**B**) genes by qPCR. ELISA assay cytokine concentration measurement of TNF-α (**C**), IL-10 (**D**) concentration in cell culture supernatant. Statistics are denoted as ns being no significant, ** *p* ≤ 0.01, *** *p* ≤ 0.001, and **** *p* ≤ 0.0001.

In the “in vitro-mimicking” model, DPSCs were seeded on 2D substrate or B-ECM hydrogel and co-cultured with macrophage RAW cells (Figure 7). In an aseptic environment, there was no obvious immune-regulatory effect of DPSCs towards RAW cells. However, when challenged with LPS, the co-culture system significantly increased IL10 and decreased TNF-α levels. However, a synergic effect of DPSC cultured on B-ECM hydrogel compared to the 2D substrate was not apparent.

### 2.8. Wound Healing Assay

**Figure 8:**
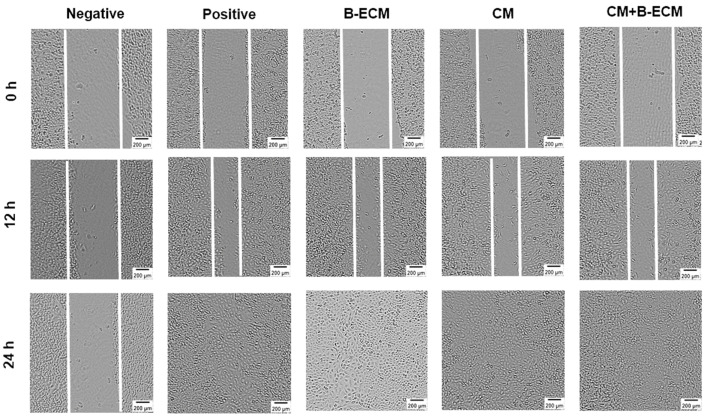
Wound healing assay of A549 cells after 12 and 24 h (scale bar = 200 µm).

The wound healing assay investigated the wound closure ability of B-ECM, CM or the combination of both in vitro, using A549 model human alveolar epithelial cells. Using ImageJ, we calculated the area of the wound scratches at 0 h. However, after 12 h, the cells started to migrate towards the wounded area in a disorderly manner, and we had difficulty using ImageJ to calculate the exact wound closure area. Nevertheless, qualitatively, after 12 h, all the experimental groups showed a comparable wound healing effect compared to the positive control and were all significantly superior to the negative control. After 24 h, the wounds were completely closed in all the groups. To provide a better demonstration, Figure 8 was processed by adjusting the threshold and using the bandpass filter functions of Image J. The original optical images can be found in the Supplementary Materials (Figure S2).

## 3. Discussion

In the use of acellular scaffolds, it is important to note that the thoroughness of decellularization is a decisive factor for ECM-based materials to elicit an anti-inflammatory macrophage/T cell host response [21]. According to the literature, the criteria for effective decellularization are: (1) less than 50 ng of double-stranded DNA per mg of dry weight, (2) DNA fragments less than 200 base pairs in length, and (3) no visible nuclei upon histologic evaluation via DAPI stains [22]. The results of decellularization in this study satisfies these standards for acellular biological materials, which indicates their safety in terms of immunogenicity.

Compared to sheet or powder forms, the hydrogel form offers advantages as it can conform to irregularly shaped defects and be applied via minimally invasive methods [23]. Although the exact mechanism for the ECM hydrogel formation process is not fully understand, it is commonly thought that it involves the self-assembly process of collagen under appropriate physiological condition of 37 °C and pH 7.4, regulated in part by the presence of glycosaminoglycans, proteoglycans, and ECM proteins [24]. In our previous study [20], neutralized digested B-ECM possessed shear thinning characteristic at 4 °C, indicating the material was injectable and could be applied in minimally invasive surgery using syringes or needles. According to rheological measurement, neutralized digested B-ECM showed solid-like behavior with a storage modulus (G′) of around 110 Pa. In the degradation test, the B-ECM hydrogel remained in solid form after 3 days of incubation in PBS. According to the literature, there are three main components of ECM materials, which are highly viscous proteoglycans (such as heparan sulfate, keratan sulfate, and chondroitin sulfate), insoluble collagen fibers, and soluble multiadhesive extracellular matrix proteins (fibronectin, laminin) [25]. The protein released at day 1 to the PBS solution were the soluble components from the B-ECM hydrogel. At days 2 and 3, no significant amount of protein was released, indicating that the B-ECM hydrogel was stable in PBS after 3 days. With excellent biocompatibility, ECM hydrogels are intensively used for loading, encapsulating and controlled delivery of cells or drugs [26]. ECM hydrogels have been shown to support the growth and differentiation ability of many different types of stem cells by providing a unique dynamic microenvironment [27]. In this study, the hydrogels fabricated from B-ECM supported the growth rate of DPSCs better than 2D control after 5 days. This may have been due to the complex biological components of the ECM materials compared to the single composition of collagen type I. In our previous study [20], the composition of B-ECM was studied using the LC-MS technique. Apart from different types of collagens, B-ECM also comprises glycoprotein and proteoglycans/GAG, fibrillin, heparan sulfate, laminin, fibulin, albumin, myosin and actin, etc. According to the literature, each individual element may play a particular role in cell proliferation and differentiation [28]. This could be the explanation for the superior supportive ability of B-ECM hydrogel towards the growth of DPSCs.

Macrophages are distributed in the human body and play a crucial function in innate immunity. When inflammatory reactions are ignited by events such as injury and infection, circulating monocytes leave the bloodstream and migrate into tissues, then differentiate into macrophages. There are two types of macrophages, which are classically activated macrophages (M1) and alternatively activated macrophages (M2). Macrophages can shift between pro-inflammatory M1 and tissue-remodeling M2, and this shift has a pivotal role in tissue regeneration [29]. In this study, ECM degradation products or the stem cell conditioned medium did not alter the functional state of macrophages, consistent with a previous study [30]. Therefore, these models could be used for studying the immuno-regulatory effect of ECM materials and MSCs on the macrophage response.

COVID-19 has become a common issue that global medical systems have had to contend with. However, effective treatments for severely ill patients have not been developed. The factor that worsens the syndromes of COVID-19 patients is cytokine release syndrome (CRS), leading to an elevated immune response. During a cytokine storm, pro-inflammatory cytokines are secreted by the immune system, which triggers an outburst of white blood cells. As a result, suppressing the cytokine storm from eliciting acute respiratory distress syndrome (ARDS) may be one of the most important factors in COVID-19 treatment [31]. According to the literature, ECM degradation products consistently downregulate pro-inflammatory and upregulate anti-inflammatory genes and proteins, although the exact mechanisms for this ability have not been fully elucidated [32,33]. Generally, it is currently agreed that many ECM components possess natural immunomodulatory domains that bind to receptors on immune cells, promoting their adhesion and adjusting their function. Moreover, the unique physical and biochemical properties of decellularized matrices affect their immunomodulatory functions [34]. In our study, the degradation products from digested B-ECM had prominent immuno-regulatory effects towards RAW macrophages under aseptic and septic conditions at both mRNA and protein levels. This ability may be due to the synergic effect of complexed ECM components towards macrophages. The major component of B-ECM is collagen, accounting for up to 50% of dried tissue [20]. Collagens I–III and XVII possess high-affinity ligands for LAIR-1 receptors on immune cells. Upon binding to the collagen, the degranulation of peripheral basophils is deterred, and thus suppresses immune cell activity. Apart from collagen, GAGs were also retained in B-ECM after decellularization [20]. Negatively charged GAG chains possess the ability to bind to positively charged immunomodulatory molecules, such as chemokines, cytokines, and growth factors, and could also influence production of these molecules [35]. Additionally, other components of ECM, such as fibrin and hyaluronic acid, have also been intensively used to investigate the potential to harness the immune system [36]. This could explain the immuno-regulatory effect of B-ECM degradation products in our study.

Presently, there are many clinical trials using MSCs of different origin for COVID-19 treatment, and DPSCs are also employed. In a clinical trial by Ye et al., DPSCs were utilized for severe cases of COVID-19 in which the safety and efficiency of DPSCs from allogeneic donors was investigated. However, the exact underlying mechanisms and results of these trials are still not fully understood [2]. In terms of COVID-19, DPSCs are superior to other sources of MSCs for the following reasons: (1) DPSCs can be easily isolated in a less invasive method from discarded teeth and with no ethical considerations; (2) DPSCs are plentifully available, easy to collect, and have efficient therapeutic effects; (3) DPSCs possess high proliferation ability, which in turn could provide sufficient cell numbers within a short time length. Moreover, they also have multi-differentiation capacity; (4) DPSCs have immunomodulatory effects similar to other sources of MSCs. Therefore, dental pulp stem cells (DPSCs) were chosen in this study as the adjuvant to the ECM material rather than MSCs from other sources [2]. Additionally, the use of DPSCs can avoid underlying issues, such as immune rejection, and teratogenicity [37]. MSCs have been intensively utilized as a promising therapeutic strategy for the treatment of inflammation and tissue injury-related diseases because they have powerful regulatory capability towards both innate and adaptive immune response systems [38]. In a septic environment, MSCs exhibit their protective functions due to the release of soluble paracrine factors, such as IL10, PGE2, TNF-α and IL-6 [39]. MSCs can reprogram macrophages from a pro-inflammatory state to an anti-inflammatory state through the release of PGE2, causing increased secretion of IL-10 [40]. In a study of lung inflammation under sepsis conditions, Pedrazza et al. found that co-culture of MSCs from epididymal adipose tissue with RAW cells could lead to a significant decrease in TNF-α and IL-6 (pro-inflammatory cytokines) and a significant increase in IL-10 (anti-inflammatory cytokines) when the cells were challenged with LPS at different time points [41]. In our study, DPSCs had a marked effect on IL10 upregulation at mRNA level; however, at protein level, over-expression of this cytokine was not observed. Nevertheless, this result was similar to previous studies investigating the immunomodulatory effect of stem cell conditioned medium on macrophages, in which IL10 was higher than the control group at gene level but was not significantly different at the protein level [30,42]. This phenomenon may be ascribed to the time-dependent manner of cytokine secretion by macrophages [43]. In the study, the authors found that when induced with LPS, at mRNA level, human alveolar macrophages expressed the highest level of IL10 at 15 h, but at protein level, IL10 concentration was only at a peak at 24 h. This indicated that IL10 may require a longer time to express at the protein level compared to the gene level.

A549, a human alveolar epithelial cell, is commonly used as the model for wound healing assays in many studies [44,45]. In a study into the effect of bladder ECM materials (urinary bladder matrix, UBM) on bleomycin-induced pulmonary fibrosis, digested UBM exhibited improvement in wound closure compared to the control group. The authors proposed that small peptides from the degradation of laminin and collagen IV in the basement membrane increased the migration of airway epithelial cells and thus stimulated wound healing [44]. Additionally, according to the literature, the basement membrane components possess a promoting effect on wound healing in a variety of organs, by directing re-epithelialization and separating the epithelium from the interstitial connective tissue [46]. In another study, the conditioned medium (CM) from umbilical cord Wharton’s jelly-derived MSCs was employed to study the wound healing potential towards A549 [45]. The results showed that in the presence of CM, the wound was efficiently amended over 24 h compared to the control group. These positive effects may be attributed to the high concentration of proteins, including growth factors, cytokines and extracellular matrix proteins secreted into the medium by the MSCs. Many of these proteins, such as insulin-like growth factor (IGF-I), interleukin-10, transforming growth factor-β and fibroblast growth factor 2 have been shown to mediate the process of proliferation and migration of many types of cells [47]. The above-mentioned findings may be the best explanations for the results of the wound healing assay in our study, in which digested B-ECM and CM from DPSCs promoted the wound healing process of A549 with results comparable to the positive control.

However, our study is not without limitations. The study only focused on the ability of B-ECM hydrogel to support the growth of DPSCs and its immunomodulatory potential when used as a scaffold to culture DPSC under aseptic and septic conditions. In studies of the immunoregulatory potential of MSCs or ECM materials, two representative cytokines, such as TNF-α and IL10, have often been employed [30,36,48]. However, other cytokines related to immune-regulatory response, such as IL-1, IL-6, IL-8, IL-12, TGF-β, etc., or different cell types of the immune system, should be used in future research to obtain a broader picture of the effects of ECM materials, as well as of MSCs, on immunomodulatory functions. From another perspective, ECM, in the form of powder, enzymatically digested solution or hydrogel, has been intratracheally injected into the lung in many in vivo studies, with results showing positive therapeutic effects towards bleomycin-induced pulmonary fibrosis [44], pulmonary oxidant damage [49], and radiation-induced lung injury [50]. These studies highlight the possibility of injecting ECM hydrogel into the lungs in vivo with no other adverse effects, and the potential of ECM as a treatment for lung diseases. As stated at the outset, B-ECM hydrogel could be a scaffold for DPSCs to stay longer in the lungs to exert their immunomodulatory effects. Therefore, animal studies should be included in the next stage to test our theory, and to provide a better insight into the immuno-regulatory potential of ECM materials. The released molecules from B-ECM or from DPSCs into the culture medium should also be accessed using LC-MS to determine the protein profiles that contribute to the immunomodulatory effects of ECM and DPSCs.

## 4. Conclusions

B-ECM hydrogel served as an excellent scaffold to culture DPSCs compared to 2D culture. Moreover, the degradation products of B-ECM materials exhibited a potential immunomodulatory effect towards macrophages under inflammatory conditions. In light of the results of our study, it is hypothesized that B-ECM hydrogel could anchor stem cells to stay longer in the lungs to express their immune-regulatory functions, and that the degradation products of the B-ECM hydrogel itself assist in the macrophage immune response. The use of digested B-ECM and CM from DPSCs favored wound healing effects on alveolar epithelial cells. Altogether, therefore, the combination of B-ECM hydrogel material and DPSCs could represent a promising in vitro model to study adjuvant therapies in cytokine-related lung diseases, before conducting in vivo animal experiments.

## 5. Materials and Methods

### 5.1. Tissue Decellularization

The pig bladders were collected from 100 to 110 kg pigs from the Taoyuan slaughterhouse (Taoyuan, Taiwan). Three to five native bladders were lyophilized and ground into pieces. The tissues were decellularized using our previously published method [51]. In brief, the tissues were treated with 1% (*w*/*v*) sodium dodecyl sulfate (SDS, Sigma-Aldrich, St. Louis, MO, USA) for 24 h, then incubated in 10% fetal calf serum (FCS, GE Healthcare Life Sciences) for an additional two days, sterilized with 0.1% (*v*/*v*) peracetic acid (PAA) (Ginyork, Taiwan) for 2 h, and finally washed with sterilized PBS. The decellularized bladders (B-ECM) were lyophilized and stored at −20 °C until use.

### 5.2. Decellularization Confirmation

The efficacy of the decellularization process was confirmed in terms of dsDNA quantification (*n* = 3), using a Quant-iT™ PicoGreen™ dsDNA Assay Kit (Thermo-Fisher Scientific, Waltham, MA, USA), DAPI staining (6-diamidino-2-phenylindole, Sigma-Aldrich, St. Louis, MO, USA) and electrophoresis, as previously described [51].

### 5.3. Preparation of B-ECM Degradation Products

The degradation products of ECM have been described previously [16]. The comminuted B-ECM was digested with 3 mg/mL pepsin (Sigma-Aldrich, St. Louis, MO, USA) in 0.01 N hydrochloric acid (HCl) for 48 h at room temperature, and then referred to as digested B-ECM.

### 5.4. Preparation of B-ECM Hydrogels

The preparation of B-ECM hydrogel was conducted as previously described [52]. The digested B-ECM was neutralized to pH 7.4 using 0.1 N NaOH and diluted to 6 mg/mL using 10× and 1× PBS at 4 °C. The neutralized digested B-ECM was incubated at 37 °C for 1 h to achieve complete gelation.

### 5.5. B-ECM Hydrogels In Vitro Degradation

The in vitro degradation of B-ECM was investigated according to a previous study with some modifications [53]. Briefly, 250 µL of 6 mg/mL neutralized B-ECM was added in a well of 24-well plates and incubated at 37 °C for 1 h to form hydrogels. 1 mL of PBS was added on top of the hydrogels and incubated at 37 °C. After 1, 2, and 3 days, the supernatants were collected and the degraded products of B-ECM hydrogels inside the PBS were measured by BCA assay (*n* = 3). Hydrogel degradation was determined by calculating the quantity of proteins released in the supernatant and calculated by protein loss/ECM weight %.

### 5.6. Mesenchymal Stem Cell Verification

Human dental pulp stem cells (DPSC) were obtained from Lonza (PT-5025). The stemness of human dental pulp stem cells (DPSCs) (p. 16) was confirmed by flow cytometry as previously described [30]. The expression of positive (CD105) and negative (CD33) markers of mesenchymal stem cells was analyzed, using a BD FACS Canto II flow cytometer (BD Biosciences, San Jose, CA, USA). Briefly, cells were stained with PE anti-human CD105 antibody (323205, Biolegend, San Diego, CA, USA) and APC anti-human CD33 antibody (303407, Biolegend, San Diego, CA, USA), following the manufacturer’s instructions.

### 5.7. DPSC Conditioned-Medium Collection

After stemness confirmation, 3 × 10^5^ DPSCs (p. 16) were seeded in a 75 T flask and cultured to 90% confluence in standard medium (SM) of DMEM containing 10% fetal bovine serum (FBS) and 1% penicillin for 2 days. The conditioned medium (CM) was then collected, filtered through a 0.22 µm filter, and stored at −80 °C until use.

### 5.8. Culture of RAW 264.7 with Digested B-ECM Supplement

A total of 2.5 × 10^4^ and 5 × 10^4^ RAW 264.7 mouse macrophages (Academia Sinica, Taiwan) were seeded in a 24-well plate (for macrophage response assays) and a 6-well plate (for immunomodulatory assay), respectively, using standard medium (SM), supplemented with 0.25 mg/mL neutralized digested B-ECM, which was referred to as the “B-ECM” group. The control group was RAW cells cultured in SM only, which was referred to as the “2D” group.

### 5.9. Culture of RAW 264.7 with Conditioned-Medium Supplement

A total of 2.5 × 10^4^ RAW 264.7 mouse macrophages were cultured in a 24-well plate, using 50% (*v*/*v*) standard medium (SM) and 50% (*v*/*v*) conditioned medium (CM) from DPSCs for 24 h as previously described [30], which was referred to as the “CM” group in this context. The control group was RAW cells cultured in SM only (2D group).

### 5.10. DPSC Growth Rate on Hydrogel Surface

The B-ECM hydrogels were prepared as described above. A total of 2.5 × 10^4^ DPSCs (p. 16) were seeded on the surface of the hydrogels. Cells were cultured in standard medium (SM). Control groups were cells cultured on tissue-culture treated plates (2D group). The cell growth rate of DPSC was measured by Alamar blue assay (Bio-rad, Hercules, CA, USA) after 1, 3 and 5 days (*n* = 3).

### 5.11. “In Vitro Mimicking” Co-Culture of RAW 264.7 Mouse Macrophages with Human DPSC

DPSCs and macrophages were co-cultured in 6-well plates, using a transwell model. A total of 5 × 10^4^ RAW 264.7 cells were seeded in the upper transwell insert with pore size of 0.40 μm (SPL Life Sciences, Pocheon, Korea). In the lower well, 200 µL B-ECM hydrogels were injected and 2 × 10^5^ DPSCs (p. 16) were seeded on the hydrogel surface. This co-culture on B-ECM hydrogel was referred to as the “CO-GEL” group. Another group of DPSCs were seeded on the lower well of tissue-culture treated plates only; this co-culture on 2D substrate group was referred to as the “CO-2D” group. As a control, RAW 264.7 cells were cultured in the upper well only. This mono-culture group was referred to as the “2D” group. The cells were maintained in standard medium (SM).

### 5.12. Macrophage Response Assays

After 24 h for the supplemented culture system, Alamar blue assay, NBT test, and neutral red assay (NR) (*n* = 4) were performed to observe functional states of unstimulated RAW cells, such as viability, phagocytosis, and pinocytosis, respectively, as previously described [30]. Briefly, the Alamar blue assay for monitoring cell viability was conducted as mentioned above. The absorbance was measured at 570 nm and 600 nm and the results were presented as the percentage reduction in Alamar blue. In the NBT test, 1 mg/mL NBT solution (Nitro blue tetrazolium chloride, Sigma, St. Louis, MO, USA) was added to the wells and incubated for 1 h at 37 °C. Formed formazan deposits were dissolved with 10% sodium dodecyl sulfate (SDS) in 0.01 M hydrochloric acid (HCl) overnight. Absorbance was read at 550 nm. In the NR assay, cells were incubated with 0.1 mg/mL neutral red solution (Sigma-Aldrich, St. Louis, MO, USA) for 30 min at 37 °C. The deposited dye was then released in 1% acetic acid/50% ethanol solution and absorbance was read at 540 nm.

### 5.13. Immunomodulatory Assay

After 20 h for the supplemented culture system and 44 h for the co-culture system, the RAW cells were stimulated with 10 ng/mL of lipopolysaccharide (LPS) from *Escherichia coli* (Sigma-Aldrich, St. Louis, MO, USA) for 4 h. The negative control was cells cultured in standard medium only (unstimulated condition). After that, the media were collected and stored at −80 °C for ELISA assay, and the cells were collected for qPCR assay.

### 5.14. Real-Time Quantitative PCR (qPCR)

Total RNA of the cells was extracted using the Trizol reagent (Thermo-Fisher Scientific, Waltham, MA, USA). The RNA was synthesized into cDNA, using a SensiFAST™ cDNA Synthesis Kit (BIO-65054, Meridian Bioscience, London, UK), following the manufacturer’s instructions. Polymerase chain reaction was performed, using SensiFAST™ SYBR^®^ No-ROX Kit (BIO-98020, Meridian Bioscience, London, UK), following the manufacturer’s instructions. The primer sets for pro-inflammatory cytokines, TNF-α, anti-inflammatory cytokines, IL10, and internal control GAPDH were used as previously described [54,55]. The primer sequences can be found in the Supplementary Materials (Table S1). Real time qPCR reactions were performed using a LightCycler^®^ 480 System (Roche, Rotkreuz, Switzerland) (*n* = 3). The relative expression levels for each gene were normalized to the 2D group under the unstimulated condition using LightCycler^®^ 480 Software (v1.5, Roche, Rotkreuz, Switzerland) as previously described [56].

### 5.15. Enzyme-Linked Immunosorbent Assays (ELISA)

The medium collected in the immunomodulatory assay was used for measuring the cytokines secreted by RAW 264.7 under unstimulated and LPS-stimulated conditions. The level of TNF-α and IL10 was measured using Mouse TNF alpha Uncoated ELISA (88-7324) and Mouse IL-10 Uncoated ELISA (88-7105) (Thermo-Fisher Scientific, Waltham, MA, USA), respectively (*n* = 3). Both assays were conducted according to the manufacturer’s instructions.

### 5.16. Wound Healing Assay

The wound healing assay was performed as previously described [45]. Briefly, 2.5 × 10^4^ human alveolar epithelial A549 cells (National Defense Medical Center, Taiwan) were seeded in 24-well plates and cultured to confluence. A 200 µL pipette tip was used to scratch the monolayer. After wounding, the cell debris was removed by washing with PBS. Wounded monolayers were then replenished with standard medium (positive control), serum free-medium (negative control), standard medium with 0.25 mg/mL B-ECM (B-ECM group), standard medium with 50% CM (CM group), or standard medium with 50% CM and 0.25 mg/mL B-ECM (CM+B-ECM group). The wound was observed under an inverted microscope (IMT-2, Olympus, Tokyo, Japan). The wound area after 0, 12 and 24 h was then measured (*n* = 4) by ImageJ (National Institutes of Health, Bethesda, MD, USA).

### 5.17. Statistical Analysis

All data are presented as mean ± standard deviation. One-way ANOVA with Sidak’s multiple comparison test was performed using GraphPad Prism v8.4.3 (La Jolla, CA, USA) to determine statistical significance between experimental groups. Differences were considered statistically significant at * *p* ≤ 0.05, ** *p* ≤ 0.01, *** *p* ≤ 0.001, and **** *p* ≤ 0.0001.

## Data Availability

The data presented in this study are available in the article.

## Supplementary Materials

The following supporting information can be downloaded at: https://www.mdpi.com/article/10.3390/gels8030187/s1, Figure S1. Optical image of DPSC cultured on 2D substrate and on B-ECM hydrogel (scale bar = 200 µm). Figure S2: Optical image of wound healing assay of A549 cells after 12 and 24 h; Table S1: Primer sequence.

## References

[1] Mahendiratta S., Bansal S., Sarma P., Kumar H., Choudhary G., Kumar S., Prakash A., Sehgal R., Medhi B. (2021). Stem cell therapy in COVID-19: Pooled evidence from SARS-CoV-2, SARS-CoV, MERS-CoV and ARDS: A systematic review. Biomed. Pharmacother. Biomed. Pharmacother..

[2] Zayed M., Iohara K. (2020). Immunomodulation and Regeneration Properties of Dental Pulp Stem Cells: A Potential Therapy to Treat Coronavirus Disease 2019. Cell Transpl..

[3] Sharma D., Zhao F. (2021). Updates on clinical trials evaluating the regenerative potential of allogenic mesenchymal stem cells in COVID-19. NPJ Regen. Med..

[4] Yao D., Ye H., Huo Z., Wu L., Wei S. (2020). Mesenchymal stem cell research progress for the treatment of COVID-19. J. Int. Med. Res..

[5] Khatri M., Richardson L.A., Meulia T. (2018). Mesenchymal stem cell-derived extracellular vesicles attenuate influenza virus-induced acute lung injury in a pig model. Stem Cell Res..

[6] Fujita Y., Kadota T., Araya J., Ochiya T., Kuwano K. (2018). Clinical Application of Mesenchymal Stem Cell-Derived Extracellular Vesicle-Based Therapeutics for Inflammatory Lung Diseases. J. Clin. Med..

[7] Wang L., Li Y., Xu M., Deng Z., Zhao Y., Yang M., Liu Y., Yuan R., Sun Y., Zhang H. (2021). Regulation of Inflammatory Cytokine Storms by Mesenchymal Stem Cells. Front. Immunol..

[8] Chen Y.-T., Miao K., Zhou L., Xiong W.-N. (2021). Stem cell therapy for chronic obstructive pulmonary disease. Chin. Med. J..

[9] Armitage J., Tan D.B.A., Troedson R., Young P., Lam K.V., Shaw K., Sturm M., Weiss D.J., Moodley Y.P. (2018). Mesenchymal stromal cell infusion modulates systemic immunological responses in stable COPD patients: A phase I pilot study. Eur. Respir. J..

[10] Karaoz E., Kalemci S., Ece F. (2020). Improving effects of mesenchymal stem cells on symptoms of chronic obstructive pulmonary disease. Bratisl. Lek. Listy.

[11] De Oliveira H.G., Cruz F.F., Antunes M.A., de Macedo Neto A.V., Oliveira G.A., Svartman F.M., Borgonovo T., Rebelatto C.L., Weiss D.J., Brofman P.R. (2017). Combined Bone Marrow-Derived Mesenchymal Stromal Cell Therapy and One-Way Endobronchial Valve Placement in Patients with Pulmonary Emphysema: A Phase I Clinical Trial. Stem Cells Transl. Med..

[12] Frantz C., Stewart K.M., Weaver V.M. (2010). The extracellular matrix at a glance. J. Cell Sci..

[13] Fernández-Pérez J., Ahearne M. (2019). The impact of decellularization methods on extracellular matrix derived hydrogels. Sci. Rep..

[14] Bonnans C., Chou J., Werb Z. (2014). Remodelling the extracellular matrix in development and disease. Nat. Rev. Mol. Cell Biol..

[15] Brown B.N., Londono R., Tottey S., Zhang L., Kukla K.A., Wolf M.T., Daly K.A., Reing J.E., Badylak S.F. (2012). Macrophage phenotype as a predictor of constructive remodeling following the implantation of biologically derived surgical mesh materials. Acta Biomater..

[16] Sicari B.M., Dziki J.L., Siu B.F., Medberry C.J., Dearth C.L., Badylak S.F. (2014). The promotion of a constructive macrophage phenotype by solubilized extracellular matrix. Biomaterials.

[17] Brown B.N., Valentin J.E., Stewart-Akers A.M., McCabe G.P., Badylak S.F. (2009). Macrophage phenotype and remodeling outcomes in response to biologic scaffolds with and without a cellular component. Biomaterials.

[18] Sadtler K., Sommerfeld S.D., Wolf M.T., Wang X., Majumdar S., Chung L., Kelkar D.S., Pandey A., Elisseeff J.H. (2017). Proteomic composition and immunomodulatory properties of urinary bladder matrix scaffolds in homeostasis and injury. Semin. Immunol..

[19] Mendibil U., Ruiz-Hernandez R., Retegi-Carrion S., Garcia-Urquia N., Olalde-Graells B., Abarrategi A. (2020). Tissue-Specific Decellularization Methods: Rationale and Strategies to Achieve Regenerative Compounds. Int. J. Mol. Sci..

[20] Kao C.-Y., Nguyen H.-Q.-D., Weng Y.-C., Hung Y.-H., Lo C.-M. (2021). Evaluating the Effect of Tissue Selection on the Characteristics of Extracellular Matrix Hydrogels from Decellularized Porcine Bladders. Appl. Sci..

[21] Keane T.J., Londono R., Turner N.J., Badylak S.F. (2012). Consequences of ineffective decellularization of biologic scaffolds on the host response. Biomaterials.

[22] Badylak S.F., Gilbert T.W. (2008). Immune response to biologic scaffold materials. Semin. Immunol..

[23] Spang M.T., Christman K.L. (2018). Extracellular matrix hydrogel therapies: In vivo applications and development. Acta Biomater..

[24] Brightman A.O., Rajwa B.P., Sturgis J.E., McCallister M.E., Robinson J.P., Voytik-Harbin S.L. (2000). Time-lapse confocal reflection microscopy of collagen fibrillogenesis and extracellular matrix assembly in vitro. Biopolymers.

[25] Yue B. (2014). Biology of the extracellular matrix: An overview. J. Glaucoma.

[26] Claudio-Rizo J., Delgado J., Quintero I., Mata-Mata J., Mendoza-Novelo B. (2018). Decellularized ECM-Derived Hydrogels: Modification and Properties. Hydrogels.

[27] Tibbitt M.W., Anseth K.S. (2009). Hydrogels as extracellular matrix mimics for 3D cell culture. Biotechnol. Bioeng..

[28] Hoshiba T., Chen G., Endo C., Maruyama H., Wakui M., Nemoto E., Kawazoe N., Tanaka M. (2016). Decellularized Extracellular Matrix as an In Vitro Model to Study the Comprehensive Roles of the ECM in Stem Cell Differentiation. Stem Cells Int..

[29] Gordon S. (2007). The macrophage: Past, present and future. Eur. J. Immunol..

[30] Stojanović S., Najman S. (2019). The Effect of Conditioned Media of Stem Cells Derived from Lipoma and Adipose Tissue on Macrophages’ Response and Wound Healing in Indirect Co-culture System In Vitro. Int. J. Mol. Sci..

[31] Musial C., Gorska-Ponikowska M. (2021). Medical progress: Stem cells as a new therapeutic strategy for COVID-19. Stem Cell Res..

[32] Dziki J.L., Huleihel L., Scarritt M.E., Badylak S.F. (2017). Extracellular Matrix Bioscaffolds as Immunomodulatory Biomaterials. Tissue Eng. Part A.

[33] Huleihel L., Dziki J.L., Bartolacci J.G., Rausch T., Scarritt M.E., Cramer M.C., Vorobyov T., LoPresti S.T., Swineheart I.T., White L.J. (2017). Macrophage phenotype in response to ECM bioscaffolds. Semin. Immunol..

[34] Taraballi F., Sushnitha M., Tsao C., Bauza G., Liverani C., Shi A., Tasciotti E. (2018). Biomimetic Tissue Engineering: Tuning the Immune and Inflammatory Response to Implantable Biomaterials. Adv. Healthc. Mater..

[35] Kang I., Chang M.Y., Wight T.N., Frevert C.W. (2018). Proteoglycans as Immunomodulators of the Innate Immune Response to Lung Infection. J. Histochem. Cytochem. Off. J. Histochem. Soc..

[36] Rowley A.T., Nagalla R.R., Wang S.-W., Liu W.F. (2019). Extracellular Matrix-Based Strategies for Immunomodulatory Biomaterials Engineering. Adv. Healthc. Mater..

[37] Lan X., Sun Z., Chu C., Boltze J., Li S. (2019). Dental Pulp Stem Cells: An Attractive Alternative for Cell Therapy in Ischemic Stroke. Front. Neurol..

[38] Barry F.P., Murphy J.M. (2004). Mesenchymal stem cells: Clinical applications and biological characterization. Int. J. Biochem. Cell Biol..

[39] Monsel A., Zhu Y.-G., Gennai S., Hao Q., Liu J., Lee J.W. (2014). Cell-based therapy for acute organ injury: Preclinical evidence and ongoing clinical trials using mesenchymal stem cells. Anesthesiology.

[40] Németh K., Leelahavanichkul A., Yuen P.S., Mayer B., Parmelee A., Doi K., Robey P.G., Leelahavanichkul K., Koller B.H., Brown J.M. (2009). Bone marrow stromal cells attenuate sepsis via prostaglandin E(2)-dependent reprogramming of host macrophages to increase their interleukin-10 production. Nat. Med..

[41] Pedrazza L., Cubillos-Rojas M., de Mesquita F.C., Luft C., Cunha A.A., Rosa J.L., de Oliveira J.R. (2017). Mesenchymal stem cells decrease lung inflammation during sepsis, acting through inhibition of the MAPK pathway. Stem Cell Res..

[42] Li Y., Gao X., Wang J. (2018). Human adipose-derived mesenchymal stem cell-conditioned media suppresses inflammatory bone loss in a lipopolysaccharide-induced murine model. Exp. Med..

[43] Chanteux H., Guisset A.C., Pilette C., Sibille Y. (2007). LPS induces IL-10 production by human alveolar macrophages via MAPKinases- and Sp1-dependent mechanisms. Respir. Res..

[44] Manni M.L., Czajka C.A., Oury T.D., Gilbert T.W. (2011). Extracellular matrix powder protects against bleomycin-induced pulmonary fibrosis. Tissue Eng. Part A.

[45] Chen J., Li Y., Hao H., Li C., Du Y., Hu Y., Li J., Liang Z., Li C., Liu J. (2015). Mesenchymal Stem Cell Conditioned Medium Promotes Proliferation and Migration of Alveolar Epithelial Cells under Septic Conditions In Vitro via the JNK-P38 Signaling Pathway. Cell. Physiol. Biochem. Int. J. Exp. Cell. Physiol. Biochem. Pharmacol..

[46] Vracko R. (1974). Basal lamina scaffold-anatomy and significance for maintenance of orderly tissue structure. Am. J. Pathol..

[47] Ionescu L., Byrne R.N., van Haaften T., Vadivel A., Alphonse R.S., Rey-Parra G.J., Weissmann G., Hall A., Eaton F., Thébaud B. (2012). Stem cell conditioned medium improves acute lung injury in mice: In vivo evidence for stem cell paracrine action. Am. J. Physiology. Lung Cell. Mol. Physiol..

[48] Saldaña L., Bensiamar F., Vallés G., Mancebo F.J., García-Rey E., Vilaboa N. (2019). Immunoregulatory potential of mesenchymal stem cells following activation by macrophage-derived soluble factors. Stem Cell Res..

[49] Wu J., Ravikumar P., Nguyen K.T., Hsia C.C., Hong Y. (2017). Lung protection by inhalation of exogenous solubilized extracellular matrix. PLoS ONE.

[50] Zhou J., Wu P., Sun H., Zhou H., Zhang Y., Xiao Z. (2020). Lung tissue extracellular matrix-derived hydrogels protect against radiation-induced lung injury by suppressing epithelial-mesenchymal transition. J. Cell. Physiol..

[51] Kao C.-Y., Nguyen H.-Q.-D., Weng Y.-C. (2020). Characterization of Porcine Urinary Bladder Matrix Hydrogels from Sodium Dodecyl Sulfate Decellularization Method. Polymers.

[52] Freytes D.O., Martin J., Velankar S.S., Lee A.S., Badylak S.F. (2008). Preparation and rheological characterization of a gel form of the porcine urinary bladder matrix. Biomaterials.

[53] Wang B., Li W., Dean D., Mishra M.K., Wekesa K.S. (2018). Enhanced hepatogenic differentiation of bone marrow derived mesenchymal stem cells on liver ECM hydrogel. J. Biomed. Mater. Res. Part A.

[54] Rodrigues Neves C.T., Spiekstra S.W., de Graaf N.P.J., Rustemeyer T., Feilzer A.J., Kleverlaan C.J., Gibbs S. (2020). Titanium salts tested in reconstructed human skin with integrated MUTZ-3-derived Langerhans cells show an irritant rather than a sensitizing potential. Contact Dermat..

[55] Kooreman N.G., de Almeida P.E., Stack J.P., Nelakanti R.V., Diecke S., Shao N.Y., Swijnenburg R.J., Sanchez-Freire V., Matsa E., Liu C. (2017). Alloimmune Responses of Humanized Mice to Human Pluripotent Stem Cell Therapeutics. Cell Rep..

[56] Tellmann G. (2006). The E-Method: A highly accurate technique for gene-expression analysis. Nat. Methods.

