# Supplementary material for: Investigating the Immunomodulatory Potential of Dental Pulp Stem Cell Cultured on Decellularized Bladder Hydrogel towards Macrophage Response In Vitro
Source: Gels. 2022 Mar 18;8(3):187. doi: 10.3390/gels8030187 (PMC8954673; doi:10.3390/gels8030187)
Supplement: Supplementary file 1 [file gels-08-00187-s001.zip › gels-1629662-supplementary.pdf]

SUPPLEMENTARY MATERIALS

Table S1. Primer sequence

Target gene	Forward primer (5′ to 3′)	Reverse primer (5′ to 3′)
**TNF-α**	GGTGCCTATGTCTCAGCCTCTT	GGTGCCTATGTCTCAGCCTCTT
**IL-10**	CGGGAAGACAATAACTGCACCC	CGGGAAGACAATAACTGCACCC
**GAPDH**	CATCACTGCCACCCAGAAGACTG	ATGCCAGTGAGCTTCCCGTTCAG

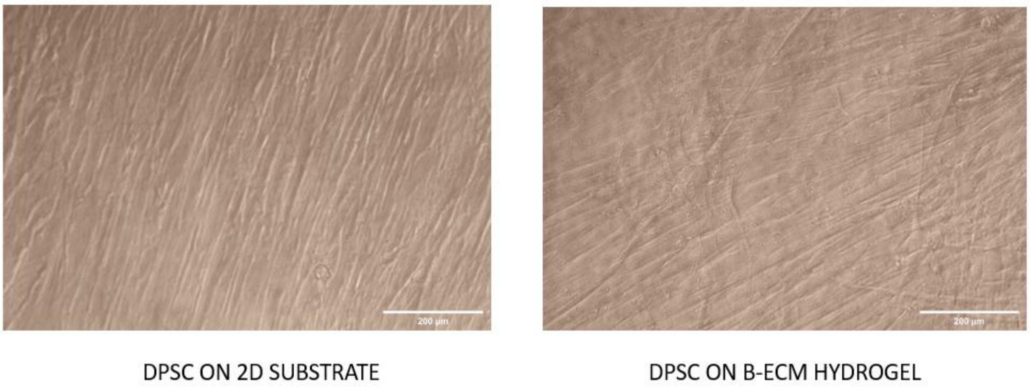

Figure S1. Optical image of DPSC cultured on 2D substrate and on B-ECM hydrogel (scale bar = 200 μm).

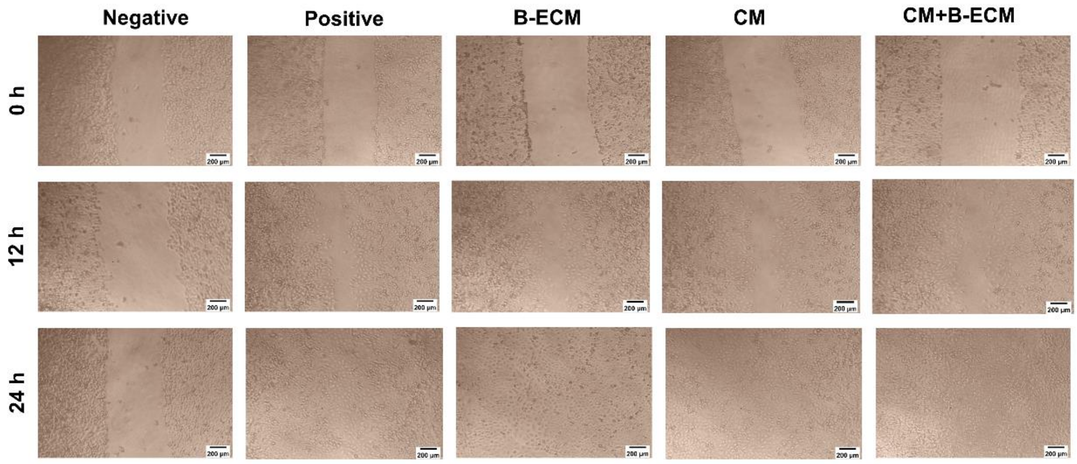

Figure S2. Optical image of wound healing assay of A549 cells after 12 and 24 h (scale bar = 200 μm).
